# Left Ventricular Summit—Concept, Anatomical Structure and Clinical Significance

**DOI:** 10.3390/diagnostics11081423

**Published:** 2021-08-06

**Authors:** Marcin Kuniewicz, Artur Baszko, Dyjhana Ali, Grzegorz Karkowski, Marios Loukas, Jerzy A. Walocha, Mateusz K. Hołda

**Affiliations:** 1 HEART-Heart Embryology and Anatomy Research Team, Department of Anatomy, Jagiellonian University Medical College, Kopernika 12, 31-034 Cracow, Poland; kuniewiczm@gmail.com (M.K.); d.ali@uj.edu.pl (D.A.); j.walocha@uj.edu.pl (J.A.W.); 2 Department of Electrocardiology, Institute of Cardiology, Jagiellonian University Medical College, 31-008 Kraków, Poland; gkarkowski@interia.pl; 3 John Paul II Hospital, 31-202 Kraków, Poland; 4 Department of Cardiology, Poznań University of Medical Sciences, 61-701 Poznań, Poland; abaszko@wp.pl; 5 Department of Anatomical Sciences, St. George’s University, West Indies, Grenada; mloukas@sgu.edu; 6 Department of Anatomy, Varmia and Mazury University, 10-082 Olsztyn, Poland; 7 Division of Cardiovascular Sciences, The University of Manchester, Manchester M13 9PL, UK

**Keywords:** left ventricular summit, cardiac anatomy, septal perforator, ventricular arrhythmia

## Abstract

The left ventricular summit (LVS) is a triangular area located at the most superior portion of the left epicardial ventricular region, surrounded by the two branches of the left coronary artery: the left anterior interventricular artery and the left circumflex artery. The triangle is bounded by the apex, septal and mitral margins and base. This review aims to provide a systematic and comprehensive anatomical description and proper terminology in the LVS region that may facilitate exchanging information among anatomists and electrophysiologists, increasing knowledge of this cardiac region. We postulate that the most dominant septal perforator (not the first septal perforator) should characterize the LVS definition. Abundant epicardial adipose tissue overlying the LVS myocardium may affect arrhythmogenic processes and electrophysiological procedures within the LVS region. The LVS is divided into two clinically significant regions: accessible and inaccessible areas. Rich arterial and venous coronary vasculature and a relatively dense network of cardiac autonomic nerve fibers are present within the LVS boundaries. Although the approach to the LVS may be challenging, it can be executed indirectly using the surrounding structures. Delivery of the proper radiofrequency energy to the arrhythmia source, avoiding coronary artery damage at the same time, may be a challenge. Therefore, coronary angiography or cardiac computed tomography imaging is strongly recommended before any procedure within the LVS region. Further research on LVS morphology and physiology should increase the safety and effectiveness of invasive electrophysiological procedures performed within this region of the human heart.

## 1. Introduction

Despite the intensive research in the anatomical sciences for the past couple of centuries, some structures within the body remain incompletely described [1,2,3]. Moreover, recent developments in invasive cardiology and electrophysiology have renewed interest in detailed cardiac anatomy [4,5]. One of the structures that are currently of special interest is the left ventricular summit (LVS). The LVS is defined as a triangular epicardial area located at the bifurcation of the left coronary artery. Recent studies are reemphasizing the importance of LVS as the source of ventricular arrhythmias with a high level of difficulty for treatment with radiofrequency ablation [6]. There are various approaches for reaching LVS arrhythmias.

Nevertheless, the proximity of the surrounding major cardiac structures might pose a risk of complications, and therefore, ablation within the LVS region may be challenging [7]. Important anatomical structures, such as surrounding coronary arteries, epicardial fat and fibrotic components, may complicate the approach [8]. Therefore, this review aims to give an extensive review of the anatomical terminology and procedural knowledge of LVS that may improve the understanding of this heart region to facilitate the performance of the radiofrequency ablation procedures.

## 2. LVS Definition

**Figure 1:**
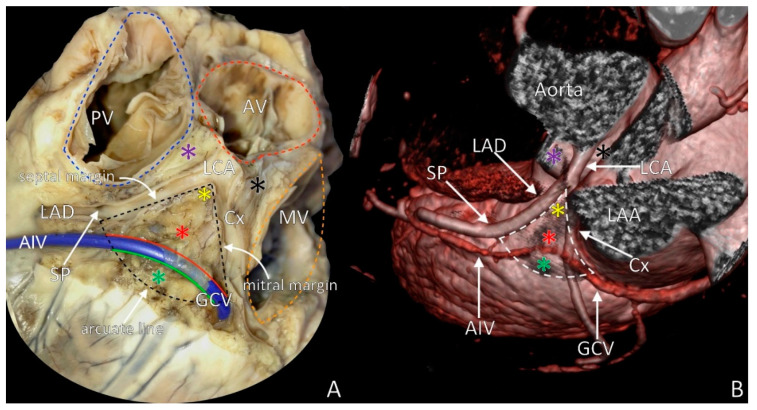
The left ventricular summit (LVS) with marked boundaries. (**A**) The LVS in heart cadaveric specimen; (**B**) LVS in medical imaging visualization (angio-computed tomography rendered image). Red asterisk = inaccessible area, green asterisk = accessible area, violet asterisk = septal summit, black asterisk = aortic–mitral continuity, yellow asterisk = apex of LVS, AIV = anterior interventricular vein, AV = aortic valve, Cx = circumflex branch of left coronary artery, GCV = great cardiac vein, LAA = left atrial appendage, LAD = left anterior descending artery, LCA = left coronary artery, MV = mitral valve, PT = pulmonary trunk, PV = pulmonary valve, SP = septal perforator.

The LVS is a triangular area located at the most superior portion of the left epicardial ventricular region, surrounded by the two branches of the left coronary artery: the left anterior interventricular artery and the left circumflex artery [9]. The triangle is bounded by septal and mitral margins (Figure 1). A delineation by an arched line, with the radius of this arc being the distance from the left coronary bifurcation artery to the first prominent left coronary artery septal perforator, represents the most inferior boundary of this arc region (triangle base). Inside this triangular area, the anterior interventricular cardiac vein (that travels through the anterior part of the atrioventricular groove) becomes the great cardiac vein that is further heading to the posterior part of the atrioventricular groove [10,11,12,13,14]. Thus, the great cardiac vein/anterior interventricular cardiac vein bisects the LVS into two distinct areas: superior area (also named the inaccessible area for radiofrequency ablation because of the significant risk of coronary vasculature damage) and inferior area (the accessible area for radiofrequency ablation, where the interventions are relatively safe) (Figure 1A) [7]. Each part of the LVS has a specific relation to the adjacent anatomical structures, and each will be described below (Figure 1).

## 3. LVS Apex

**Figure 2:**
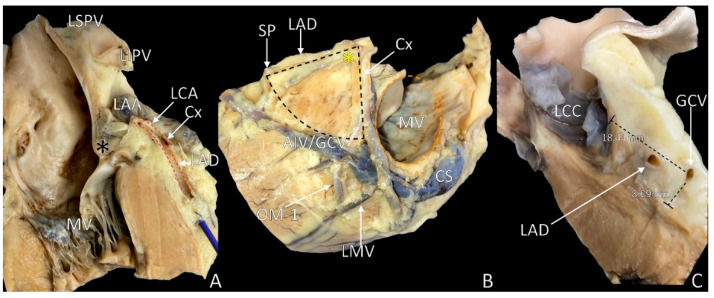
Photographs of cadaveric heart specimens showing the left ventricular summit (LVS) area with overlying epicardial adipose tissue. (**A**) Section through the septal margin of the LVS; (**B**) view of the LVS after adipose tissue removal; (**C**) section through the LVS showing the abundance of the adipose tissue. Black asterisk = aortic–mitral continuity, yellow asterisk = apex of LVS, AIV = anterior interventricular vein, CS = coronary sinus, Cx = circumflex branch of left coronary artery, GCV = great cardiac vein, LAA = left atrial appendage, LAD = left anterior descending artery, LCC = left coronary cusp, LIPV = left inferior pulmonary vein, LMV = left marginal vein, LSPV = left superior pulmonary vein, OM = obtuse marginal branch, MV = mitral valve, SP = septal perforator.

The apex of the LVS is located superiorly towards the left coronary arterial ostium. It begins at the left coronary artery’s bifurcation to the left circumflex coronary artery and left anterior descending artery. The nearest neighboring structure to the LVS apex is the aortic root, covered by a large fibrous structure—the aorto-ventricular membrane [9] (Figure 2A). The aortic root is the continuation of the left ventricle outflow tract. It occupies a central position within the heart, located to the right and posteriorly, relative to the subpulmonary infundibulum [7]. The apex into the deep of the myocardium correlates with the left aortic sinus of Valsalva, septal summit to the right and aortic–mitral continuity to the left (Figure 1). The epicardial adipose tissue and pulmonary trunk cover the LVS apex (Figure 2A). The mean distance from the LVS apex to the aortic sinus origin of the left coronary artery is approximately 10 mm, but may even reach 21 mm [15,16]. In approximately 10% of hearts, the left coronary artery trifurcates. Its third branch, the ramus intermedius, penetrates the LVS area, trespassing in the midsection over the accessible and inaccessible areas. In almost 2% of cases, the apex of the LVS cannot be defined because of the absence of the circumflex branch of the left coronary artery since it originates directly from the left sinus of Valsalva. In patients with bicuspid aortic valves, variants are even more commonly observed [16].

## 4. The Septal Margin of LVS

The anterior interventricular groove with the adjacent structures—the left anterior descending artery and anterior interventricular vein—determines the septal (or superior) boundary of the LVS [8,17,18]. The relationship between those two vessels in the majority (54.4%) of cases presents a profound intersection of the anterior interventricular vein to the left anterior descending artery [19]. Along the artery and vein, the left coronary cardiac nerve forms the left coronary subplexus that contributes to the autonomic innervation of the left ventricle [20]. The septal margin corresponds with the pulmonary trunk. Nevertheless, a few millimeters of the septal summit, or the septal aspect of LVS, are present between the anterior part of the interventricular groove and the pulmonary trunk. The distance from the left coronary artery bifurcation to the first dominant septal perforator denotes the length of the septal margin of the LVS [8]. The annulus of the pulmonary valve and pulmonary trunk is present above the LVS septal margin. These structures are overlying the LVS in the most superior aspect. The right ventricular outflow tract correlates with the lower portion of the septal margin, and various amounts of epicardial adipose tissue are present between those structures (Figure 2C). From the septal margin of LVS, the first or sometimes second diagonal branch enters the LVS region while septal perforators penetrate the ventricular septum. Rarely, the right-sided branches occur, creating preconal ring anastomosis—arterial ring of Vieussens [21]. The anterior interventricular vein enters from the anterior aspect of the interventricular groove into the LVS.

In some cases, when the most dominant septal perforator is more proximal to the left coronary artery bifurcation, the anterior interventricular vein may not cross the septal margin of the LVS but is then located more distally, outside the LVS triangle. In an uncommon variation, the anterior interventricular vein may not be present within the LVS area as it may drain to the right, towards the anterior cardiac vein [22]. The presence of the myocardial bridges over the left anterior descending artery should also be mentioned, as their presence is not insignificant within the left coronary artery tree. Nevertheless, within the anterior interventricular artery, the myocardial bridges are usually located outside the LVS area as they are much more likely to be found in the middle segment of the artery (75.3%), followed by distal (20.5%) and proximal (7.4%) segments of the left anterior interventricular artery [23,24].

## 5. Mitral Margin of LVS

The left circumflex coronary artery, together with the accompanying left lateral cardiac nerve, forms the mitral (or inferior) boundary of the LVS. In the distal sector of the mitral margin, the small section of the great cardiac vein is also present (Figure 1) [25]. In some cases, the circumflex coronary artery is accompanied by the conus vein, which trespasses under the trunk of the left coronary artery (Figure 2A,B). The length of the mitral margin is equal to the length of the septal margin. This margin neighbors close to the mitral annulus (thus the name of the margin), left fibrous trigon, left atrium and the left atrial appendage base [19]. The left atrial appendage overlaps the mitral margin, most commonly covering around 80% of its length, but this may vary depending on the left atrial appendage body type (shape) [26].

In some cases, the mitral margin’s superior aspect may be covered by the pulmonary trunk or left main pulmonary artery [15]. The first (or sometimes the second) obtuse marginal branch originates from the left circumflex coronary artery entering into the LVS from the mitral margin aspect. The great cardiac vein enters over a mitral margin of the LVS. It crosses the left circumflex coronary artery and then runs into the posterior aspect of the atrioventricular groove. Several spatial relationships between the great cardiac vein and the left circumflex coronary artery may be found, with the vein located over the artery being the most common [19]. The left phrenic nerve trespassing epicardially over the left atrial appendage and then directing to the left dome of the diaphragm may cross the mitral margin [27,28,29,30].

## 6. The Base of the LVS: Arcuate Line

The base of the LVS may be defined as a curved line, with the radius of this arc being the distance from the left coronary artery bifurcation to the first dominant septal perforator originating from the left anterior descending artery [8]. Thus, the base of the LVS creates an imaginary line over the left ventricle’s epicardial surface that may be trespassed by ramus intermedius, diagonal branches and obtuse marginal branches of the left coronary artery (Figure 2B). Moreover, in some cases, the venous system (represented by the anterior interventricular vein or great cardiac vein with tributaries) may cross the arcuate line.

**Figure 3:**
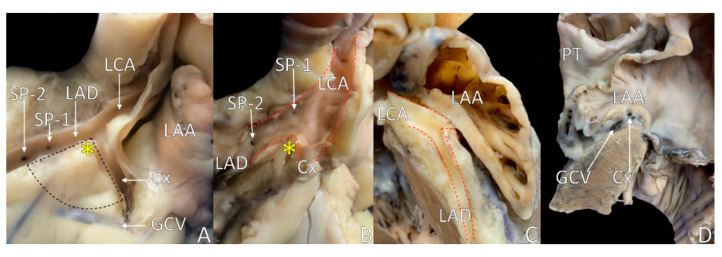
Photographs of cadaveric heart specimens showing the left ventricular summit (LVS) area with opened left coronary artery tree and overlying left atrial appendage (LAA). (**A**) Septal perforators (SPs) are visible in left anterior descending (LAD) artery with SP-1 being small and SP-2 being bigger and dominant. The great cardiac vein (GCV) is running below the SP; thus, the LVS has only an inaccessible area. (**B**) SPs located in the great proximity of the left coronary artery (LCA) ostium: the small SP-1 located in the left coronary artery (LCA) trunk and the dominant SP-2 located near the LCA bifurcation, thus defining a very small LVS area. (**C**,**D**) The LAA is located directly over the LVS area. Cx = circumflex branch of left coronary artery, PT = pulmonary trunk.

**Figure 4:**
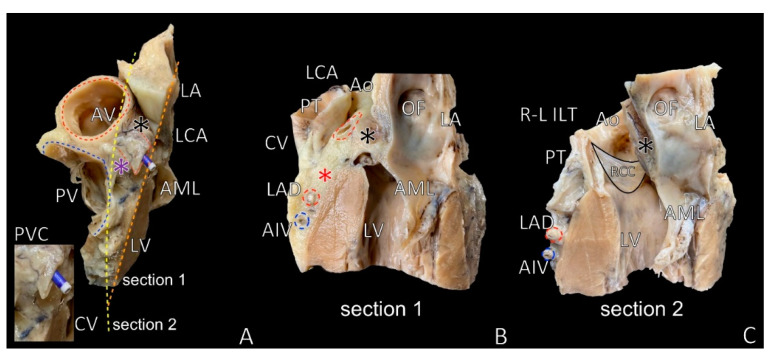
Photographs of the same cadaveric heart specimen dissected at two different levels showing spatial relationships between left ventricular summit (LVS), coronary vessels and aortic valve components. (**A**) Block of heart showing section lines through the LVS area; (**B**) Section 1, cut through the anterior interventricular groove (along the LVS septal margin); (**C**) Section 2, cut through the septal summit and the left coronary cusp of aortic valve. Violet asterisk = septal summit, black asterisk = aortic–mitral continuity, red asterisk = inaccessible area, Ao = aorta, AIV = anterior interventricular vein, AML = anterior mitral leaflet, AV = aortic valve, CV = conus vein, LA = left atrium, LAD = left anterior descending artery, LCA = left coronary artery, LV = left ventricle, OF = oval fossa, PT = pulmonary trunk, PV = pulmonary valve, PVC = posterior vein of conus, R-L ILT = right–left interleaflet triangle, RCC = right coronary cusp.

The biggest challenge in drawing the LVS base is depicting the proper septal perforator. Many septal perforators can be found in the left anterior descending artery (from one to three on the first 25 mm distance). The first anterior septal branch is usually the most prominent (40–60 mm in length) and provides the most important collateral channels. However, only 30% of normal angiograms demonstrate a large (1.5 mm in diameter or larger) first septal perforator that distally arborizes into at least four branches. In 28% of cases, the first perforator is a small artery, with a further 24% of patients having two or three arteries comparable in size. Finally, in the last 18% of the hearts, the septal perforator diffuses into multiple small septal arteries [31] (Figure 3A,B). When only one septal perforator is present in the proximal aspect of the left anterior descending artery, the definition of the LVS is indisputable, whereas when there are more perforators, the LVS definition should take the perforator with the largest diameter (Figure 4A). There is another reason for choosing the most dominant perforator that relates to available imaging methods. In cardiac computed tomography angiography, the septal perforator imaging can usually visualize a vessel larger than 1 mm in diameter. Smaller vessels visible in the macroscopic dissection might be unnoticed in clinical assessment.

## 7. LVS Size and Content

The size of the LVS may vary and does not correlate with BMI, sex or age. The distance from left coronary artery bifurcation to the first septal perforator (length of the LVS septal margin) (Figure 3) and the angle of left coronary artery bifurcation determine the LVS size. The mean size of LVS measured in computed tomography is 263 mm^2^, with the mean inaccessible area being significantly bigger than the accessible area (133 vs. 95 mm^2^). A Yamada equation calculation identifies the area with a high correlation to real LVS size [8,15].

Inside the LVS area, the presence of the great cardiac vein is almost inevitable. However, LVS may come without a coronary venous tributary when there is a short distance from the left coronary artery bifurcation to the first dominant septal perforator that excludes venous vessels from the defined LVS region (Figure 2B and Figure 3A,B). In anatomical terminology, the great cardiac vein begins at the heart’s apex. It ascends along the anterior interventricular groove to the base of the heart, while in cardiology and radiology, the initial segment of the great cardiac vein (from the apex of the left ventricle to the LVS area) is named the anterior intraventricular vein [22]. By definition, the anterior intraventricular vein originates at the lower or middle third of the anterior interventricular groove, follows the groove adjacent to the left anterior descending artery and angulates laterally toward the heart’s base to form the great cardiac vein [7,32]. The point of transition between the anterior interventricular vein and the great cardiac vein lies inside the LVS, and arguably, it is a significant source of epicardial idiopathic ventricular arrhythmias [33].

The relationships between the great cardiac vein and the coronary arteries within the LVS region vary between individuals [19,34,35]. There are four main aspects of the GCV course relative to the left coronary branches: (1) side of initial course relative to the left anterior interventricular artery, (2) superficial or deep crossing of the left anterior interventricular artery from a right side initial course, (3) proximal or distal crossing of the circumflex branch of the left coronary artery and (4) superficial or deep crossing of the circumflex branch of the left coronary artery. From these aspects, Bales et al. present 12 theoretical relationship combinations [36]. Studies report that the great cardiac vein is found either on the right or left side of its related artery in the interventricular groove, more often crossing the left anterior interventricular artery superficially. The relationships of the great cardiac vein to the circumflex artery are predominantly proximal (close relative to the apex of the LVS). Approximately 60% of the great cardiac veins crossed superficially to the circumflex artery in most studies [22,37]. It is interesting to note that when the great cardiac vein crossed the left anterior interventricular artery superficially, it more often crossed the circumflex artery superficially and that when the great cardiac vein crossed the left anterior interventricular deeply, it more often crossed the circumflex artery deeply. Figure 2B presents the rarest possible variation with deep crossing to left anterior interventricular and circumflex artery in the distal relation.

The coronary vessel system lying in the LVS region is sunken in the relatively abundant epicardial adipose tissue. The distance from the epicardium to the surface of the left ventricular myocardium is thus significant (up to 10 mm). The thickness of the adipose tissue may vary significantly between individuals and between different regions of the LVS (Figure 2) [38,39]. For example, the amount of epicardial adipose tissue along the arcuate line is significantly smaller than in apical aspects of the LVS (Figure 2). This distance might impact ventricular potential amplitude recorded from the great cardiac vein if the epicardial adipose tissue is sufficiently thick. Moreover, the thick adipose tissue layer may have a pro-arrhythmogenic activity and negatively influence the success rate of electrophysiological procedures performed within the LVS [40,41,42].

## 8. LVS Nourishment and Innervation

The myocardium in the LVS area receives the arterial blood from perforating arteries branching off from the proximal segments of left coronary artery branches. The venous collection drains into the coronary sinus (via anterior coronary veins into the anterior interventricular vein or great cardiac vein) from the lower aspect of LVS and via noncoronary sinus tributaries from the apical aspect and septal side of LVS [43,44,45]. The left or right superior septal veins drain the superior third of the interventricular septum (ventricular outflow tracts). The left superior septal vein, the longest and largest intramural venous channel, can reach 2–3 mm in diameter [22] and can be used in interventional procedures [46,47,48,49]. The venous drainage from the septal summit is most commonly into the right atrium via the intramural sinus [50], also known as veins of Vieussens or communicating vein [51]. This also involves veins that drain the adipose tissue that cover the conus arteriosus [43]. An important fact is that this region is abundant in venous anastomoses with the axillary orientated circle embracing the entire heart, consisting of the coronary sinus–great cardiac vein–small cardiac vein–conus vein, and further intercommunicating veins [22].

The epicardial region of LVS is covered by the fibers of the intrinsic cardiac nervous autonomic system [52,53,54], gathering nearly 6% of all ganglionated plexuses of the heart [20,55]. These plexuses are embedded in the epicardial adipose tissue. The first portion of the left coronary ganglionated subplexus with preganglionated nerves is densely distributed nearby the pulmonary trunk and ascending aorta in septal summit region. Postganglionated nerves extend into anterior, lateral and (in part) posterior walls of the left ventricle. Moreover, thin short postganglionated nerves proceed on the interior surface of the left atrial appendage along the atrial branches of the circumflex branch of the left coronary artery [20]. The subendocardially located Purkinje network that originates from the left bundle branch’s anterior division most likely activates the LVS region. The anterior division of the left bundle branch proceeds toward the base of the superolateral papillary muscle of the mitral valve. It is formed of many fine strands coursing anteriorly to the free wall [56,57,58]; thus, this segment is most likely to support the LVS region. However, both networks from anterior and posterior divisions of the left bundle branch are widely interconnected [59,60]. Another argument for anterior division distribution in the LVS region is the possibility of mapping and ablating the left anterior fascicle from the right aortic sinus [61].

## 9. LVS Accessibility and Ablations

Over 12% of ventricular arrhythmias from the left ventricle have a source over the epicardial surface of the left ventricle [8]. The most common electrocardiographic template for LVS arrhythmias is V2 lead pattern break qrs [62]. The ablation of ventricular arrhythmias from the LVS is challenging and requires various approaches. Another pattern of outflow tract ventricular arrhythmia is with abrupt R-wave transition in the V3 lead with its source in the septal margin of the LVS [17].

Access to the LVS is required when the ventricular arrhythmia source is localized in this particular area. The coronary venous system plays an essential role in the LVS division into the superior and inferior aspects. The superior aspect of the LVS, because of the dense vasculature in which damage may be life-threatening, is defined as an inaccessible area. It is also recognizable as the triangle of Brocq and Mouchet [63,64,65,66,67]. The difference may be found in both the terminology and boundaries of both triangles, where the first dominant septal perforator marks the LVS base.

In contrast, the trigon of Brocq and Mouchet base is marked by the point where the anterior interventricular vein crosses the anterior interventricular groove. The Brocq and Mouchet trigon has five main variations dependent on vessel crossings and their relations in heart groves, while the LVS area is independent of such variants [63,64]. The inaccessible area is almost triangular, closed at the bottom by the venous system. Usually, the superior aspect dominates in size over the accessible area, and frequently, the LVS may be constituted only by inaccessible area (Figure 1, Figure 2B and Figure 3A). The inaccessible area contents include proximal branches from coronary vessels, small veins and a thick layer of epicardial adipose tissue covered by the left atrial appendage and pulmonary trunk.

The anterior interventricular artery/great cardiac vein line and LVS arcuate line, the accessible part of the LVS, are present. The accessible area is mostly irregular in shape, with the presence of coronary vessels originating from the inaccessible area and anterior coronary veins draining into the coronary venous system. In addition, the density of coronary branches is significantly lower, and epicardial adipose tissue is less abundant than in inaccessible areas. Thus, it is named “accessible”. Moreover, the accessible LVS area is often uncovered by the left atrial appendage with epicardial approach possibility [68].

Because the LVS is an epicardial region, the approach to the LVS may be indirect or direct. The indirect method uses the adjacent structures to achieve closeness to the earliest activation point exiting the arrhythmia source. Because of a lack of such reasonable access points, these were defined as the vertices of the “Bermuda triangle” [69,70,71]. However, delivery of the proper radiofrequency energy to the arrhythmia source, avoiding at the same time the coronary artery damage, may be a challenge [70,72,73]. Therefore, coronary angiography or cardiac computed tomography imaging is strongly recommended before any procedure within the LVS region [74,75,76,77,78,79,80,81].

The nearest relation to the LVS apex or septal aspect of the LVS is the left coronary cusp of the aortic valve or right–left interleaflet triangle [7,17,82] (Figure 4). The septal margin approach is possible to perform from the left coronary cusp of the pulmonary valve and pulmonary trunk in the superior aspect and from the right ventricular outflow tract in the inferior aspect of allied anatomical conditions (Figure 4). The septal perforators may also reach the LVS myocardium near the septal margin. The mitral margin may be accessible from the left atrial appendage or great cardiac vein (Figure 3) [33]. A mid aspect of the LVS can be attainable from the venous system: the great cardiac vein, the anterior interventricular vein, the communicating vein, the conus vein and septal venous perforators, or recently proposed left atrial appendage, which covers nearly 75% of the LVS region [15,33,51] (Figure 3 and Figure 4).

Nevertheless, access from the coronary venous vasculature may be challenging because of the complex anatomy of the cardiac venous system. Although the distance between the coronary veins and left ventricular myocardium may be significant (up to 20 mm), the range of the ThermoCool electrodes allows reaching the arrhythmia source from the epicardial aspect, making ablation successful. In addition, recent long-term observations from ventricular ablations in the LVS region using a bipolar technique show reduction in unattainable arrhythmias [83].

Access from the endocardial region toward the epicardial aspect of the LVS is almost impossible because of the thickness of the left ventricular myocardium. Only the subvalvular aspect of the left ventricle endocardium is thin enough to process the energy to the LVS (Figure 4) [17,84].

The direct approach to the LVS provides access through the pericardial sac. This access also has a limitation because of thick epicardial adipose tissue and the coronary vessel density. The only reachable part of LVS via direct epicardial aspect is an accessible area. The superior portion of LVS is dangerous for pericardial penetration. It increases the risk of major and minor complications, such as intrapericardial bleeding, coronary artery stenosis, delayed tamponade and incidental right ventricle puncture [85,86,87,88,89]. Table 1 summarizes possible approaches to the LVS area.

The overview of publications focusing on ablations of ventricular arrhythmias arising from the LVS region with reported success and complication rates of procedures is presented in Table 2.

## 10. Septal Summit/Septal Aspect of the LVS

Next to the LVS concept, the second idea of the so-called “septal summit” was proposed. The septal summit is the most superior part of the interventricular septum, located near the LVS septal margin, more toward the pulmonary trunk. The septal summit extends from the ventriculo-aortic junction down to the first dominant septal perforator (Figure 1). The structures neighboring the septal summit are the right–left interleaflet trigon from the top, the left pulmonary sinus from the right aspect and the anterior interventricular groove’s content from the lateral side. Above the apex of the LVS (under the left coronary artery), the septal summit corresponds with aortic–mitral continuity.

In contrast to the LVS, the septal summit cannot be reached from the left coronary aortic cusp, the left ventricular endocardial aspect or the left atrial appendage. It is worth emphasizing that the septal summit is central to the parasternal long-axis view in transthoracic heart ultrasound examination [90]. The above-mentioned right–left interleaflet trigon is adjacent to the mid-posterior septal aspect of the right ventricular outflow tract [91]. Within the septal summit, the conus vein and posterior veins of the cone, also known as communicating veins, can be present (Figure 4A) [51].

## 11. Conclusions

Providing systematic and comprehensive anatomical descriptions and proper terminology in the LVS region may facilitate the exchanging of information among anatomists and electrophysiologists, increasing knowledge of this cardiac region. We postulate that the most dominant septal perforator (not the first septal perforator) should characterize the LVS definition. Abundant epicardial adipose tissue overlying the LVS myocardium may affect arrhythmogenic processes and electrophysiological procedures within the LVS region. The LVS is divided into two clinically significant regions: accessible and inaccessible areas. Rich arterial and venous coronary vasculature and a relatively dense network of cardiac autonomic nerve fibers are present within the LVS boundaries. Although the approach to the LVS may be challenging, it may be executed indirectly using the surrounding structures. Further research on LVS morphology and physiology should increase the safety and effectiveness of invasive electrophysiological procedures performed within this region of the human heart.

## Figures and Tables

**Table 1:** List of approaches to the LVS and possible ablation coverage in the individual regions.

Approach to LVS	Reachable LVS Region	References
Right ventricular outflow track	Septal margin, right aspect of LVS accessible area, lower right septal summit	[12,70]
Pulmonary trunk/left pulmonary artery	Septal margin, right aspect of LVS inaccessible area, higher right septal summit	[6,73]
Aorta–left sinus of Valsalva/left coronary cusp	Septal summit, apex of LVS, aortic–mitral continuity	[70,71,73,75,88]
Aorta–L-R inter leaflet trigon	Septal summit, apex of LVS	[17,71,75,89]
Left atrial appendage	Mitral margin of LVS, accessible and inaccessible area (depends on the morphology and coverage of appendage)	[68,70,83]
Great cardiac vein/anterior interventricular vein	Mitral margin of LVS, between accessible and inaccessible areas (depends on the course of venous system)	[8,33,51,72,86,88,89]
Epicardial–subxiphoid access	Accessible area of LVS from septal to mitral margin	[8,38,51,70,72,73,89]

**Table 2:** Reported success and complication rates of LVS ventricular arrhythmia ablation procedures.

Study	LVS Access (if Specified)	Total Number of Cases	Number of Successful Cases	Number of Complications
Obel, 2006 [33]	Total GCV	5	5	0
Daniels, 2006 [72]	Total	11	11	0
	GCV	9		
	Epi	2		
Yamada, 2008 [75]	Total	44	44	1
	LCC	24		
	RCC	14		
	NCC	1		
	R-L ILT	5		
Kumagai, 2008 [73]	Total	45	40	0
	AMC	3		
	MA	8		
	LCC/RCC	32		
	Epi	2		
Sacher, 2010 [85]	Total Epi	136	64	8
Yamada, 2010 [8]	Total	27	22	0
	GCV	14		
	Epi	4		
Frankel, 2014 [12]	RVOT	2	2	0
Santangeli, 2015 [38]	Total Epi	23	5	no data
Yamada, 2015 [86]	Total	64	58 (only inaccesabile area failure)	0
	GCV	36		
	AMC	28		
Marai, 2016 [71]	Total	10	10	0
	R-L ILT	5		
	AIV-GCV	1		
	NCC	1		
	LCC	2		
	RCC	1		
Hayashi, 2017 [70]	Total	12	7	0
	RVOT	7		
	LCC	1		
	AIV	2		
	Epi	2		
Yamada, 2017 [87]	not specified	229	212	no data
Komatsu, 2018 [51]	Total	31		
	GVC	14	10	1
	other	17	17	0
Benhayon, 2018 [68]	LAA	1	1	0
Yakubov, 2018 [70]	LAA	1	1	0
Candemir, 2019 [88]	not specified	21	15	0
Liao, 2020 [17]	R-L ILT	20	16	0
Igarashi, 2020 [83]	not specified	18	16	3
Chung, 2020 [89]	Total	238	199	7
	GCV	91		
	EPI	6		
	RL ILT	139		

RL ILT = right–left interleaflet region, GCV = great cardiac vein, Epi = epicardial approach, RVOT = right ventricular outflow track, AMC = aortic–mitral continuity, LCC = left coronary cusp, NCC = noncoronary cusp, RCC = right coronary cusp, AIV = anterior interventricular vein, MA = mitral annulus.

## Data Availability

The data presented in this study are available on request from the corresponding author.
